# Endoscopically assisted laparoscopic local resection of gastric tumor

**DOI:** 10.1186/1756-0500-6-410

**Published:** 2013-10-12

**Authors:** Christoforos Kosmidis, Christoforos Efthimiadis, Georgios Anthimidis, Kalliopi Vasileiadou, Thomas Stavrakis, Georgia Ioannidou, Georgios Basdanis

**Affiliations:** 1 Department of Surgery, Interbalkan European Medical Center, 10 Asklipiou street, Thessaloniki, Pylaia 57001, Greece; 2 Department of Obstetrics, Interbalkan European Medical Center, Thessaloniki, Greece; 3 Department of Radiology, “Agios Pavlos” General Hospital, 161 Ethnikis Antistaseos street, Thessaloniki 55134, Greece

**Keywords:** Laparoscopic, Endoscopic, Rendezvous, Tumor, Gastric, Resection

## Abstract

**Background:**

Minimally invasive procedures have been applied in treatment of gastric submucosal tumors. Currently, combined laparoscopic - endoscopic rendezvous resection (CLERR) emerges as a new technique which further reduces operative invasiveness.

**Case presentation:**

A-57-year-old female patient presented with epigastric pain. She was submitted to gastroscopy, which revealed a tumor located at the angle of His. Biopsy specimens demonstrated a leiomyoma. The patient underwent endoscopically assisted laparoscopic resection of the tumor. The operative time was 45 minutes. Diagnosis of leiomyoma was confirmed by the final histopathological examination. The patient had an uneventful postoperative recovery and was discharged on the 2^nd^ postoperative day.

**Conclusion:**

Combined laparoscopic and endoscopic rendezvous resection appears as a promising alternative minimally invasive technique. It offers easy recognition of the tumor, regardless of location, safe dissection, and full thickness resection with adequate margins as well as less operative time.

## Background

Minimally invasive surgery has become the gold standard in numerous procedures, including those associated with gastric disease. Laparoscopic approach has been applied in resection of gastric submucosal tumors. Still, combined laparoscopic - endoscopic rendezvous resection (CLERR) presents currently as a groundbreaking technique of gastric tumor resection.

We present herein a case of endoscopically assisted laparoscopic resection of a gastric intraluminal leiomyoma.

## Case presentation

**Figures 1:**
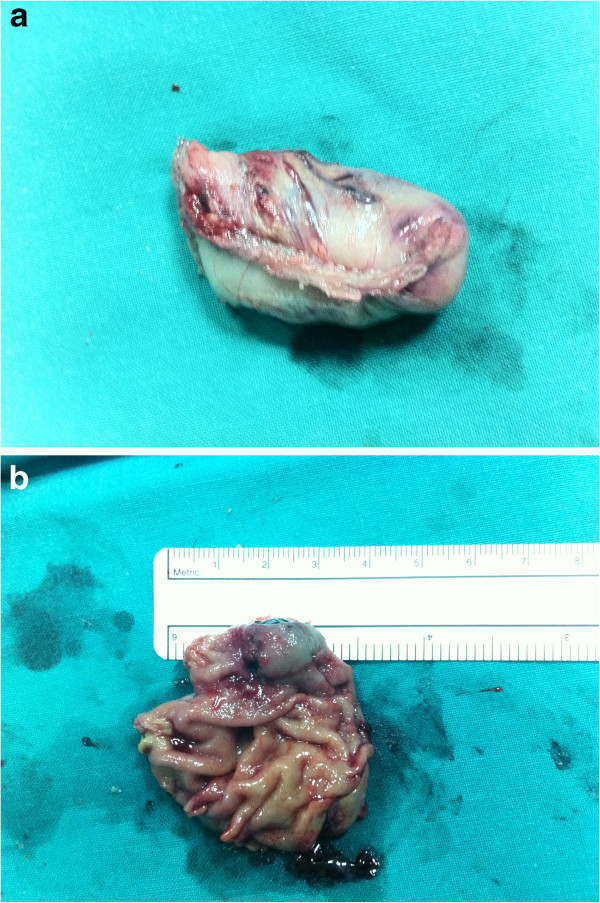
a,b. Specimen of the tumor mass.

A-57-year-old female patient presented with epigastric pain. She was submitted to gastroscopy, which showed a tumor located at the angle of His. Biopsy specimens demonstrated the benign nature of the tumor, identifying it as leiomyoma. The patient underwent endoscopically assisted laparoscopic resection of the tumor along the lesser curvature, with adequate resection margins (2 cm) (Figures 1a, 1b). The operative time was 45 minutes. Diagnosis of leiomyoma was confirmed by the final histopathological examination. The patient had an uneventful postoperative recovery and was discharged on the 2^nd^ postoperative day.

### Operative technique

**Figure 2:**
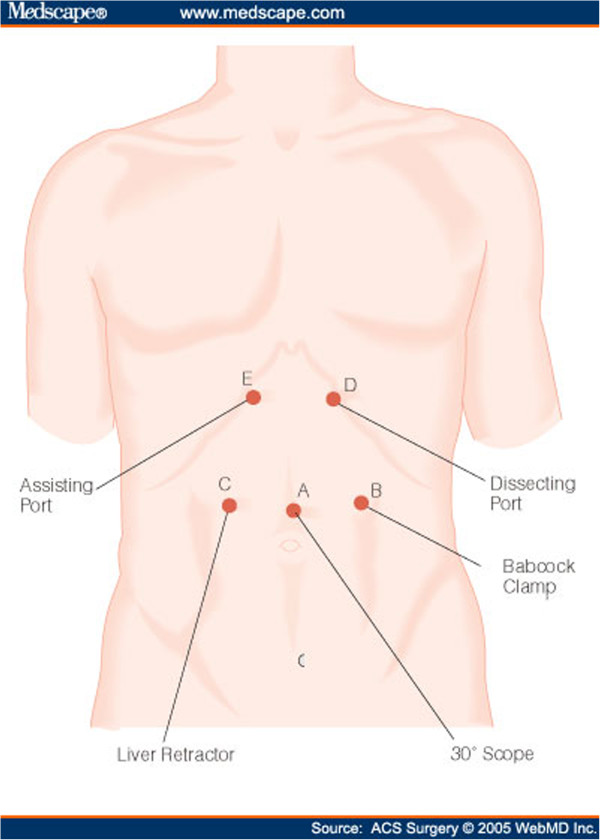
Trocar placement.

**Figure 3:**
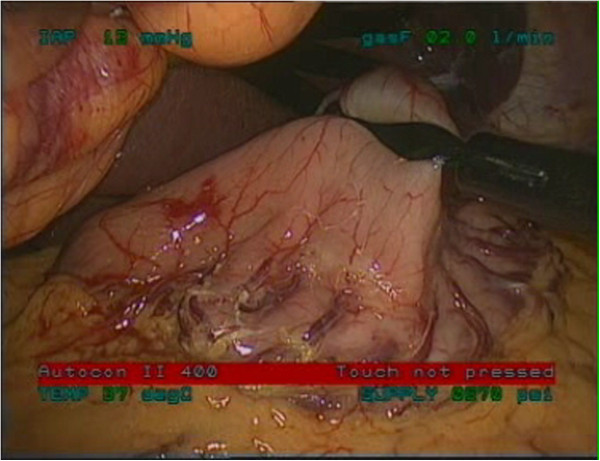
Lift of the lesion with an atraumatic grasper.

**Figure 4:**
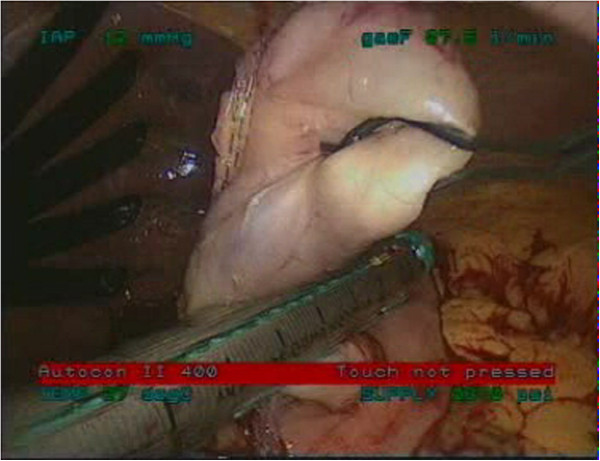
Excision of the lesion using Endo GIA stapler.

**Figure 5:**
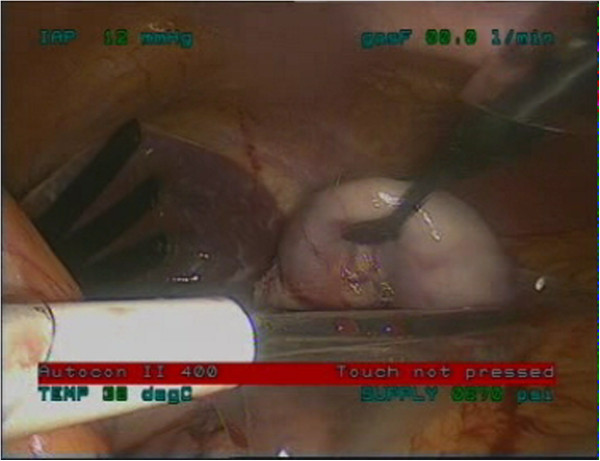
Retrieval of the specimen with a laparoscopically inserted endobag extractor.

Under general anesthesia, the patient was placed in supine position. Both endoscopic and laparoscopic monitors were arranged near the head of the patient so that the surgeon could watch both of them. The first trocar was inserted in the midline, one third of the distance from the umbilicus to the xiphoid process, using the Hasson technique, for insertion of a laparoscopic 30^0^ camera. Then, four trocars were inserted, one in each abdominal quadrant, as shown in Figure 2. The lesion had been marked preoperatively by endoscopic tattooing with India ink. The exact position of the tumor was constantly localized intraoperatively via endoscopic transillumination. The tumor was grasped with an atraumatic grasper, dissected and excised within adequate margins using Endo GIA stapler (Figures 3, 4). Careful hemostasis was carried out and the specimen was retrieved in a laparoscopically inserted endobag extractor (Figure 5).

## Conclusions

Combined laparoscopic - endoscopic rendezvous resection is a new treatment method for gastric submucosal tumors. Yet, careful patient selection, accurate preooperative diagnosis and operative strategy are required. Parameters taken into account should include size and histological type of the tumor, depth of gastric wall invasion and expansion.

Specifically, small (diameter 1–2 cm) locally limited, benign gastric mucosal tumors may be considered for CLERR. Additionally, this method is indicated for certain premalignant tumors and small locally limited submucosal gastrointestinal stromal tumors (GISTs). In fact, GISTs usually present as local gastric tumors without any infiltration of regional lymph nodes and consequently CLERR is clearly indicated in this respect [1]–[3].

On the other hand, CLERR is absolutely contraindicated for locally advanced and metastatic tumors, which should be managed using an open method [1,2]. For that reason preoperative evaluation using endoscopic ultrasound, transabdominal ultrasound and/or computed tomography scan is necessary [4]–[8].

Although endoscopic mucosa resection is nowadays quite acceptable for the treatment of early gastric cancer [6,9]–[12], CLERR emerges as an approach with the same or even more satisfactory results [6,13]–[15]. Nevertheless, these results can not be evaluated yet due to limited number of series.

Nevertheless, endoscopy appears to be valuable in guiding laparoscopic gastric resection. The gastric tumor is marked preoperatively using endoscopic tattooing and the exact position of the tumor is constantly localized intraoperatively via endoscopic transillumination. Finally surgical removal is confirmed by the endoscopist.

In general, CLERR offers the common advantages of minimally invasive approach (less pain, less inflammatory response, faster recovery, shorter hospital stay, less hospital charges, better quality of life) along with easier recognition and dissection of the tumor, regardless of its location, ensuring less operative time and complete haemostasis. It renders full thickness resection with adequate margins leading to therapy or at least a reliable histologic analysis which may guide further therapeutic decisions [5,15,16].

In conclusion, combined laparoscopic and endoscopic rendezvous resection appears to be a quite promising method with the benefits of minimally invasive surgery. In any case, careful patient selection, correct preoperative diagnosis and appropriate operative planning are required.

## Consent

“Written informed consent was obtained from the patient for publication of this Case report and any accompanying images. A copy of the written consent is available for review by the Editor of this journal”.

## Abbreviations

CLERR: Combined laparoscopic - endoscopic rendezvous resection; GISTs: Gastrointestinal stromal tumors.

## Competing interests

The authors declare that they have no competing interests.

## Authors’ contributions

CK was the main surgeon. CE supervised the paper writing. GA was the assistant surgeon. KV wrote the main manuscript. TS wrote the supplementary information. GI gave technical support and conceptual advice. All authors read and approved the final manuscript.
